# An Unclassified Disease

**Published:** 1893-01

**Authors:** 


### An Unclassified Disease.

"I hear," said Mrs. Parvenue, "that Mr. Willows' son took the diploma at Yale last year. I always said Yale was an awful unhealthy city."

